# Kin discrimination and outer membrane exchange in *Myxococcus xanthus*: Experimental analysis of a natural population

**DOI:** 10.1371/journal.pone.0224817

**Published:** 2019-11-27

**Authors:** Sarah M. Cossey, Yuen-Tsu Nicco Yu, Laura Cossu, Gregory J. Velicer

**Affiliations:** 1 Institute for Integrative Biology, Department of Environmental Systems Science, ETH Zürich, Switzerland; 2 Department of Environmental Microbiology, Eawag, Switzerland; Academia Sinica, TAIWAN

## Abstract

In some species of myxobacteria, adjacent cells sufficiently similar at the adhesin protein TraA can exchange components of their outer membranes. The primary benefits of such outer membrane exchange (OME) in natural populations are unclear, but in some OME interactions, transferred OM content can include SitA toxins that kill OME participants lacking an appropriate immunity gene. Such OME-dependent toxin transfer across *Myxococcus xanthus* strains that differ only in their *sitBAI* toxin/antitoxin cassette can mediate inter-strain killing and generate colony-merger incompatibilities (CMIs)–inter-colony border phenotypes between distinct genotypes that differ from respective self-self colony interfaces. Here we ask whether OME-dependent toxin transfer is a common cause of prevalent CMIs and antagonisms between *M*. *xanthus* natural isolates identical at TraA. We disrupted *traA* in eleven isolates from a cm-scale soil population and assayed whether *traA* disruption eliminated or reduced CMIs between swarming colonies or antagonisms between strains in mixed cultures. Among 33 isolate pairs identical at *traA* that form clear CMIs, in no case did functional disruption of *traA* in one partner detectably alter CMI phenotypes. Further, *traA* disruption did not alleviate strong antagonisms observed during starvation-induced fruiting-body development in seven pairs of strains identical at *traA*. Collectively, our results suggest that most mechanisms of interference competition and inter-colony kin discrimination in natural populations of myxobacteria do not require OME. Finally, our experiments also indicate that several closely related laboratory reference strains kill some natural isolates by toxins delivered by a shared, OME-independent type VI secretion system (T6SS), suggesting that some antagonisms between sympatric natural isolates may also involve T6SS toxins.

## Introduction

Kin discrimination among microbes can be defined phenomenologically as differential expression (or effects) of behaviour across interactants as a function of genetic relatedness [1–3]. Such behaviors include secretion of toxins to which a producer cell is genetically resistant but to which victim cells are not [4, 5] and preferential co-aggregation with genetically similar cells [1, 6]. Another form of microbial kin discrimination is boundary formation at colony interfaces that are specific to encounters between distinct genotypes, or colony-merger incompatibility (CMI, [1]). Because the evolutionary significance of social interactions hinges on the relatedness of interactants [7, 8], kin-discriminatory behaviors in both microbes [9–11] and animals [12–16] are often interpreted in the context of kin-selection theory [2]. However, because evolutionary forces other than kin selection can cause kin-discriminatory behaviors to evolve [1, 17], the ultimate causes of such behaviors among naturally-evolved microbes are often unclear.

Genetically determined CMI is a major form of microbial kin discrimination in which colonies of distinct bacterial genotypes meet through motility-driven swarming and generate a border phenotype different from the phenotypes of self-self colony borders. CMIs were first described in *Proteus mirabilis* [18] but have since been observed in other species, including *Myxococcus xanthus* [1, 19], *Bacillus subtilis* [20] and *Pseudomonas aeruginosa* [21]. CMIs are potentially important in the social evolution of motile bacteria because they can reflect the outcome of genotype x genotype interactions affecting fitness. For example, CMIs between natural isolates of *M*. *xanthus* are associated with reduced co-aggregation into shared fruiting-body groups at colony interfaces [1].

CMIs can be generated by a variety of mechanisms, both across and within species. In *P*. *mirabilis*, some inter-colony boundaries are due to effector toxins delivered by the type VI secretion system (T6SS) [22–24]. In *B*. *subtilis*, mechanisms include outer-membrane alterations that likely influence susceptibility to antimicrobials and mutations in contact-dependent growth-inhibition loci such as *wapAI* [25]. Experimental populations of *M*. *xanthus* readily evolve CMIs by a variety of genetic mechanisms [1] and toxin delivery by both outer membrane exchange (OME) [26, 27] and a T6SS [28] can generate CMIs involving relevant mutants of this species.

Myxobacteria exhibit several complex social behaviors, including coordinated group motility [29, 30] while hunting other microbes [31] and multicellular fruiting-body development in response to starvation [30, 32]. Additionally, in the model species *M*. *xanthus*, experiments showing that motility-related proteins can be transferred between cells revealed a process now known as outer membrane exchange, or OME [33, 34]. Specifically, the motility defect of a mutant unable to produce the protein Tgl, which facilitates formation of an outer-membrane-channel for type IV pili [35], was found to be rescued by inter-cellular transfer of Tgl from a *tgl*^*+*^ strain [33]. Tgl transfer was later found to occur by a contact-dependent mechanism requiring both of the co-operonic genes *traA* and *traB* [34]. A TraA/TraB protein complex facilitates OME by homotypic interaction between cell-surface-bound TraA, which appears to act as an adhesin [34, 36]. TraA-mediated OME causes cells to fuse their outer membranes and exchange large amounts of cellular material [34]. OME occurs on relatively solid surfaces but apparently not on soft, moist surfaces or in liquid [34, 36, 37].

The *traA/traB* operon is present in a majority of myxobacterial species examined to date but has not been found outside the myxobacteria [38]. However, genomes representing more than one third of all myxobacterial genera examined to date lack this operon. These include some species, such as *Chondromyces crocatus*, known to engage in complex fruiting-body formation [38]. Consistent with this finding, deletion of *traA* from *M*. *xanthus* was not found to significantly affect the major social traits of social motility or fruiting-body development in this species [34]. Thus, OME is not essential for the most prominent social behaviors of myxobacteria.

TraA has diversified into a variety of functionally distinct allotypes across the myxobacteria [38]. These include several allotypes within *M*. *xanthus* that are incompatible for OME due to dissimilarity in the PA14 hyper-variable domain of TraA [3, 36]. CMI patterns among natural isolates of *M*. *xanthus* [3] and experimentally-evolved strains [1] suggest that divergence at TraA does not contribute to CMIs. TraA has been hypothesized to be a greenbeard trait that preferentially directs cooperative benefits of OME between cells with functionally compatible TraA proteins [36]. It has been further speculated that a major group-level benefit might derive from reduction of physiological heterogeneity among neighboring cells (or, phrased inversely, an increase in group-level homeostasis) due to OME [39]. However, the selective forces responsible for the maintenance of the *traA/traB* operon in some myxobacterial species remain to be clarified [3, 26, 39–44].

In contrast to the possibility that OME may mediate cooperative benefits, it has become clear that, like contact-dependent growth inhibition in other species [45], OME has the potential to mediate strong antagonistic interactions. Dey *et al*. discovered that a polyploid prophage (Mx alpha) present in many *M*. *xanthus* strains produces SitA toxins that can transfer across cells by TraA-dependent OME and kill OME participants lacking a cognate immunity gene [46]. Wielgoss *et al*. (2016) subsequently localized this prophage to an ~150 kb region of the *M*. *xanthus* genome that is exceptionally polymorphic with respect to gene content among the Tübingen cm-scale isolates [47]. Patterns of gene content in this highly polymorphic region were found to correlate with CMI-allotype categories previously defined among a subset of cm-scale isolates from Tübingen, Germany [19]. It was thus proposed that distinct OME-transferrable toxins might be encoded in the highly polymorphic region by some strains and contribute to inter-strain antagonisms in local natural populations [47].

Toxin production that is asymmetric across colony borders is expected to generate CMIs. Consistent with this, Vassallo *et al*. [26] found that colonies of isogenic strains expressing distinct *sitBAI* toxin/immunity cassettes generate CMIs at colony interfaces due to OME-mediated killing. They further presented data suggesting that mutants of a lab reference strain incapable of OME or Sit-toxin production may be less competitive against OME-compatible natural isolates than is the parental reference strain. In light of these results, it was again suggested that OME-toxin transfer may be involved in naturally evolved forms of interference competition and CMIs.

Wielgoss *et al*. (2018) examined this hypothesis in a comparative analysis of *M*. *xanthus* natural isolates from the Tübingen cm-scale population with respect to patterns of CMIs, antagonisms and TraA diversity among isolate pairs [3]. Almost all previously documented strong antagonisms among these cm-scale strains [19, 48] were found to occur between genotypes predicted to be incompatible for OME due to dissimilarity at TraA, implicating OME-independent mechanisms. OME-independent CMIs among these strains were also common, as all strain pairs predicted to be OME-incompatible at TraA exhibited clear CMIs. Overall, patterns of TraA variation in that focal population predict that most randomly selected isolate pairs should be incompatible for OME at TraA [3], in turn suggesting that most latent CMIs and antagonisms among all theoretically possible pairs of natural isolates do not involve OME.

Nonetheless, Wielgoss *et al*. (2018) also found that a large fraction of cm-scale strain pairs identical at *traA* exhibit clear CMIs that could potentially be caused by OME-delivered toxins [3]. Diversity in natural *M*. *xanthus* populations is highly structured, such that relatedness between strains increases with decreasing distance between them [47, 49]. Thus, genetically distinct neighbors likely to interact in the soil may often be sufficiently similar at TraA to be compatible for OME [3]. It is thus relevant to test whether CMIs or antagonisms between such TraA-similar natural isolates from the same local population generally involve OME.

Here we ask whether disruption of *traA*—and hence debilitation of OME—in cm-scale natural isolates reduces or eliminates CMI boundaries at colony interfaces with other isolates that share the same *traA* allele. We further document several severe antagonisms between *traA-*identical strains and test whether *traA* disruption alleviates those antagonisms. Finally, having discovered in the course of our experiments that lab reference strains inhibit growth of natural isolates in an OME-independent manner, we test whether these antagonisms are caused by a T6SS carried by the reference strains.

## Materials and methods

### Strains and primers

The two primary lab reference strains used in this study are GJV1 and DK101 (Table 1). GJV1 is a close derivative of DK1622 [50], differing by only five mutations [51]. GJV1 is highly proficient at both mechanisms of motility employed by *M*. *xanthus* (S-motility and A-motility, A+S+) and fruiting-body development [51, 52]. DK101 is a mutant of strain FB [29] that is defective at type IV pili-mediated S-motility but has a functional A-motility system (A+S-)[53]. DK101 has three Mx-alpha prophage repeats that encode SitA toxins transferrable by OME and corresponding antitoxins [26, 46]. DK1622 was generated from DK101 through UV mutagenesis and Mx8 phage transduction that restored functional S motility [50]. During this process DK1622 lost two Mx-alpha repeats and the associated toxin and antitoxin genes, which resulted in susceptibility to OME-mediated killing by DK101 [46]. Also, DK101 colonies expand only very slowly on a hard-agar surface compared to GJV1 and the natural isolates used in this study (S1 Fig). For these reasons, DK101 was utilized to test for loss of *traA* function in relevant mutants, as described further below.

**Table 1:** Strains.

Strain	Description	Reference
DK101	A+S- (proficient at A-motility, defective at S-motility)	[29]
GJV1	A+S+ DK1622 derivative	[51, 52]
GJV1*traA*	*Mxan_6895* knock-in mutant	[3]
GJV1*traA*-S	*Mxan_6895* knock-in mutant	This study
GJV1*traB*	*Mxan_6898* knock-in mutant	This study
A88	Natural isolate	[54]
A88*traA*	*Mxan_6895* knock-in mutant	This study
SA3437	DK1622 *ΔvgrG1* (*Mxan_4800*)	[55]
SA5700	DK1622 *ΔvgrG2* (*Mxan_5573*)	[55]
SA5701	DK1622 *ΔtagF* (*Mxan_4805*)	[55]
SA5707	DK1622 ΔT6SS (*Mxan_4800*-*Mxan_4813*)	[55]
SA5712	DK1622 *ΔvgrG1 ΔvgrG2*	[55]
A00	Natural isolate	[54]
A23	Natural isolate	[54]
A30	Natural isolate	[54]
A31	Natural isolate	[54]
A32	Natural isolate	[54]
A44	Natural isolate	[54]
A46	Natural isolate	[54]
A60	Natural isolate	[54]
A72	Natural isolate	[54]
A93	Natural isolate	[54]
A00*traA*	*Mxan_6895* knock-in mutant	This study
A23*traA*	*Mxan_6895* knock-in mutant	This study
A44*traA*	*Mxan_6895* knock-in mutant	This study
A60*traA*	*Mxan_6895* knock-in mutant	This study
A72*traA*	*Mxan_6895* knock-in mutant	This study
A60Δ*traA*	*Mxan_6895* in-frame deletion	This study
A23rif^R^	Rifampicin resistant A23	[19]
A07	Natural isolate	[54]
A26	Natural isolate	[54]
A47	Natural isolate	[54]
A96	Natural isolate	[54]
A07*traA*	*Mxan_6895* knock-in mutant	This study
A47*traA*	*Mxan_6895* knock-in mutant	This study
A96*traA*	*Mxan_6895* knock-in mutant	This study
A47rif^R^	Rifampicin resistant A47	[19]
A96rif^R^	Rifampicin resistant A96	[19]
A17	Natural isolate	[54]
A38	Natural isolate	[54]
A65	Natural isolate	[54]
A73	Natural isolate	[54]
A38*traA*	*Mxan_6895* knock-in mutant	This study
A01	Natural isolate	[54]
A33	Natural isolate	[54]
A45	Natural isolate	[54]
A01*traA*	*Mxan_6895* knock-in mutant	This study
Top10	*E*. *coli* strain for plasmid construction	Invitrogen

*M*. *xanthus* strains are grouped by predicted TraA compatibility for OME, with each compatibility group containing an identical *traA* allele separated by a line.

Additional strains are natural isolates of *M*. *xanthus* originally isolated from a 16 x 16-cm soil patch in Tübingen, Germany [54] and *traA* disruption or deletion mutants of those isolates (Table 1). Social compatibilities and competitive abilities of some of those isolates have been characterized [3, 19, 48], as have the phylogenetic and predicted functional relationships of TraA sequences across the entire cm-scale isolate set [3]. The subset of strains used in this study was selected to represent five of the TraA compatibility groups predicted by Wielgoss *et al*. [3]. These compatibility groups were predicted based on phylogenetic context of the relevant Tübingen cm‐scale isolates relative to isolates previously tested for OME [3, 36, 40].

Primers used in this study are listed in Table 2. In a previous study, *traA*-disruption mutants defective at OME were generated by plasmid integration of a 543-bp insert [34]. Here, two primer pairs (Table 2) were used to amplify a slightly larger (788 bp) region of *traA* in GJV1 and the natural isolates. This larger region was used to disrupt *traA* because it allowed generation of the plasmid constructs for all natural isolates with common primers aligned to homologous sequence. Plasmid integration was verified using primer M13 or M13r (depending on the orientation of the plasmid used when generating the *traA* mutants) in combination with an additional primer (GV763) designed to bind upstream of the plasmid integration site.

**Table 2:** Plasmids and primers.

Plasmid	Description	Reference
pCR-Blunt	Cloning/integrative vector	Invitrogen
pBJ113	Allele exchange plasmid with kan^R^ and *galK* genes	[56]
pDP3	pCR2.1 containing *traB* insertion cassette	[34]
pCR_A88*traA*	Contains a 788-bp fragment of *Mxan_6895* amplified using primers GV751 and GV753	This study
pCR_A00*traA*	Contains a 788-bp fragment of *Mxan_6895* amplified using primers GV752 and GV755	This study
pCR_A23*traA*	Contains a 788-bp fragment of *Mxan_6895* amplified using primers GV752 and GV755	This study
pCR_A44*traA*	Contains a 788-bp fragment of *Mxan_6895* amplified using primers GV752 and GV755	This study
pCR_A60*traA*	Contains a 788-bp fragment of *Mxan_6895* amplified using primers GV752 and GV755	This study
pCR_A72*traA*	Contains a 788-bp fragment of *Mxan_6895* amplified using primers GV752 and GV755	This study
pCR_A60Δ*traA* pBJ_A60Δ*traA*	In-frame deletion of *Mxan_6895* (amino acid residues 13–695) generated using primers GV765, GV766, GV767, and GV768	This study
pCR_A07*traA*	Contains a 788-bp fragment of *Mxan_6895* amplified using primers GV752 and GV755	This study
pCR_A47*traA*	Contains a 788-bp fragment of *Mxan_6895* amplified using primers GV752 and GV755	This study
pCR_A96*traA*	Contains a 788-bp fragment of *Mxan_6895* amplified using primers GV752 and GV755	This study
pCR_A38*traA*	Contains a 788-bp fragment of *Mxan_6895* amplified using primers GV751 and GV753	This study
pCR_A01*traA*	Contains a 788-bp fragment of *Mxan_6895* amplified using primers GV752 and GV755	This study
**Primer**	**Sequence**	
GV751	5’ TCACTGTCTTGTCGGTGTGCCTC 3’	
GV752	5’ TCACTGTCCTGGCGGTGTGCCTC 3’	
GV753	5’ AAGAAGGTGTGCCTCCCGCCTGC 3’	
GV755	5’ TTGCCGTAGGAGAGGAAGCTTCC 3’	
GV756	5’ GCCGGTTGATGACCTGATACGG 3’	
GV763	5’ GTGGGAGATATCCCTCATTG 3’	
GV765	5’ AGCGCTACCACGTGGACCCC 3’	
GV766	5’ TTTCAAAGCCCCGCAACAATG 3’	
GV767	5’TGTTGCGGGGCTTTGAAATTCCTGCTGCTGCTCGCCGCG 3’	
GV768	5’ CCTCCAGGTTGGCGCCGCCC 3’	
M13	5’ GTAAAACGACGGCCAG 3’	
M13r	5’ CAGGAAACAGCTATGAC 3’	

### Pre-assay culture conditions

Strains were inoculated from frozen stocks onto CTT [57] 1.5% agar plates and allowed to grow for 3–5 days at 32° C and 90% relative humidity. Strains were subsequently transferred into 8 mL of liquid CTT and grown overnight until turbid while shaking at 32° C, 300 rpm, diluted in 8 mL CTT and again grown overnight. On the day each experiment was initiated, cultures were grown to mid-log phase, centrifuged at 12,000 rpm for 5 minutes and then re-suspended in TPM liquid buffer [57] to a density of ~5 x 10^9^ cells/mL for use in assays of swarming inhibition, kin discrimination, and developmental competition.

### Construction of *traA/B*-disruption mutants

An internal 788-bp region of *traA* was amplified from natural isolates and GJV1 with primers listed in Table 2 to generate the corresponding pCR-*traA* plasmids (Table 2). Additional primers were used to amplify an internal 543-bp fragment of the GJV1 *traA* allele to create a *traA* knock-in mutant similar to the one used by Pathak *et al*. for comparison [34]. Amplicons were gel extracted and ligated into the pCR-Blunt vector (Invitrogen), which confers resistance to kanamycin. Plasmids (pCR_*traA*) putatively including the relevant *traA* fragment were verified by EcoRI digestion and sequencing. The plasmids constructed with the 543 and 788-bp fragments of the GJV1 *traA* allele are labelled pCR_*traA*-S and pCR_*traA*, respectively.

The natural isolates and GJV1 were transformed with the *tra*A plasmids to generate corresponding *tra*A-disrupted mutants. GJV1 was also transformed with pDP3, the *traB-*disruption plasmid used by Pathak *et al*. 2012 [34]. The integration of the pCR_*traA*, pCR_*traA*-S, and pDP3 plasmids results in a mero-diploid with one allele truncated at the 3'-end and the other at the 5'-end. The disrupted mutants generated by pCR_*traA* contain the complete PA14 highly variable region of TraA while the pCR_*traA*-S mutant only possesses a portion of PA14. Cell lysates of *traA* and *traB* mutants were used in colony PCR to verify transformants. Advantage GC2 polymerase was used for the verification and the standard manufacturer recommended protocol was followed.

### In-frame deletion of *traA*

An in-frame deletion of *traA* was created in the A60 background, one of the natural isolates belonging to the largest TraA recognition group among the Tübingen isolates. Primers were designed to amplify two fragments that span approximately 500 bp upstream and downstream of *traA* in A60 that overlap by 18 bp. The two PCR products were used as the template for a sequence overlapping extension (SOE) reaction to generate a 1-kb fragment (equivalent to an in-frame deletion of TraA amino-acid residues 13–695). The in-frame deletion product was then cloned into the pCR-Blunt (Invitrogen) vector to generate pCR_Δ*traA*. The fragment was then excised from the plasmid using EcoRV and BamHI, gel purified, and cloned to the HincII-BamH1 linearized pBJ113 vector to create the plasmid pBJ_Δ*traA*.

The A60 *traA* in-frame deletion was constructed by transformation with pBJ_Δ*traA* and a previously described galactose-selection allele-exchange protocol [56]. Putative in-frame deletion clones were verified by PCR amplification and sequencing.

### Colony-merger incompatibility assays

Compatibility or incompatibility for self-self-like colony merger was determined by spotting pairs of 10 μL cell suspensions (at ~5 x 10^9^ cells/ml) at a distance of 10 (most cases) or 7.5 mm (a few cases for slow-swarming strains) on a 0.1% CTT 1.5% agar plate. Spots were allowed to dry completely and plates then were incubated at 32° C with 90% relative humidity for 5–6 days. After incubation, the presence of CMI demarcations was determined visually relative to self-self controls by previously described criteria [1, 3, 47]. Each natural isolate and the corresponding *traA* mutant were tested for CMIs with every other strain belonging to the same predicted TraA recognition group. Self-self control encounters were also done for each strain simultaneously with self-nonself assays. For this and all other assays, at least three biological replicates initiated on different days were performed.

### Swarming-inhibition assay

GJV1, all natural isolates and all *traA* mutants were assayed for swarming inhibition by DK101 in experiments similar to those of Dey *et al*. [46]. Each strain was mixed at a 1:1 ratio or a 1:9 ratio with DK101 (with the latter in the majority) from cell suspensions at ~5 x 10^9^ cells/ml. 10 μL aliquots of both the mixed cultures and pure cultures of each strain were spotted on a CTT 1.5% agar plate with 2 mM CaCl_2_. Plates were incubated at 32° C with 90% relative humidity for 5–6 days. After incubation, swarming distance was measured for all monocultures and DK101 mixes as the average distance of colony expansion along several radii from the time of inoculation to the time of measurement.

The percentage-swarm expansion of each focal strain mixed with DK101 relative to its swarm expansion in pure culture was calculated as:

$$\frac{S[focal:DK101]-S[GJV1:DK101]}{S[focal]-S[GJV1:DK101]}\times 100$$

where *S* is the distance swarmed by a pure culture of a focal strain *S*[*focal*] or a mixed culture of a focal strain or GJV1 with DK101 (*S*[*focal*:*DK101*] or *S*[*GJV1*:*DK101*], respectively). The percentage-relative swarm expansion was calculated within each replicate for each strain pair and averaged across replicates. Because DK101 kills GJV1, mixed cultures of these strains expand at a rate indistinguishable from pure colonies of DK101.

### Killing assay

We tested whether DK101 and GJV1 kill the kanamycin-resistant strain A60*traA* in mixed cultures with an agar-flipping assay initiated in the same manner as the swarming-inhibition assays described above, except A60*traA* was mixed with each paired strain at a 1:2 (A60*traA*:partner) ratio. After four days of incubation, the agar containing the initially mixed swarm was cut out using a scalpel and flipped upside-down onto a CTT 1.5% agar plate containing 40 μg/mL kanamycin. Killing was determined by the absence of growth on the kanamycin-containing plate. Controls of A60*traA* mixed with its parent A60, A60*traA* in pure culture and GJV1*traA* mixed with each of GJV1 and DK101 were also performed with the same assay.

Five T6SS mutants [55] that were constructed in the DK1622 background were similarly tested for their ability to kill A60*traA*. One of the mutants, SA5701, retains T6SS activity [55]. The other mutants have deletions in either *vgrG1*, *vgrG2*, both *vgrG1* and *vgrG2*, or the entire intact T6SS (*Mxan*_4800-*Mxan_*4813).

To confirm that the lack of growth by A60*traA* observed after mixing with some strains in the flipped-agar assay described above was due to killing of A60*traA* by the respective aggressor strain, we performed a colony-forming unit (CFU) recovery experiment to evaluate the survival rate of A60*traA* after 24 hours of pairwise co-incubation with strains of interest. DK101, GJV1, A60, A60*traA*, SA5701(*ΔtagF*), and SA5707(*ΔT6SS*) were cultured as described under ‘Pre-assay culture conditions’. The kanamycin-resistant strain *A60traA* was mixed in the minority (1:2 ratio) with each of the other strains (all kanamycin-sensitive) listed above. 10 μL of the mixed cell suspension were spotted onto a CTT 1.5% agar plate supplemented with 2 mM CaCl_2_, and incubated at 32° and 90% relative humidity for 24 hours. The entire inoculum was harvested with a sterile scalpel followed by serial dilution and plating in CTT soft agar with and without kanamycin. Colonies were counted after six days of incubation at 32°C and 90% relative humidity.

### Developmental antagonism assays

Previously described rifampicin-resistant mutants of A23, A47 and A96 [19] and *traA* mutants of the same strains (Table 1) were mixed at a 1:99 ratio with other unmarked natural isolates prior to initiation of starvation-induced fruiting-body development. In all cases, the unmarked isolate in each pairing was previously predicted to belong to the same TraA-compatibility category as the parent of each focal mutant [3]. Specifically, A23rif^R^ and A23*traA* were mixed in the minority with A30, A72 and A93, A47rif^R^ and A47*traA* were mixed with A96 and A96rif^*R*^ and A96*traA* with A07, A26, and A47. Additionally, A23rif^R^ was mixed in the minority with A00, A32, A46 and A60 and A47rif^R^ was mixed in the minority with A07 and A26. All strains also simultaneously underwent development in pure culture.

## Results

### Plasmid disruption of *traA* eliminates OME-mediated toxin transfer

To test whether TraA-mediated toxin delivery plays a major role in *M*. *xanthus* kin discrimination, we constructed plasmid-insertion mutants in which plasmid integration created mero-diploids containing two non-functional copies of *traA*. We modified a previously described swarming assay [46] to confirm loss of *traA* or *traB* function upon integration of relevant plasmids into strain GJV1, the genome sequence of which is nearly identical to the published DK1622 sequence [51]. Both DK1622 and GJV1 derive from strain DK101 and possess a functional S-motility system (involving type IV pili), which DK101 lacks. Both DK1622 and GJV1 lack Mx-alpha toxin/antitoxin cassettes carried by DK101 and it has been shown that DK1622 is killed in an OME-dependent manner by strains carrying the same Mx-alpha toxin cassettes as DK101 [46].

**Fig 1:**
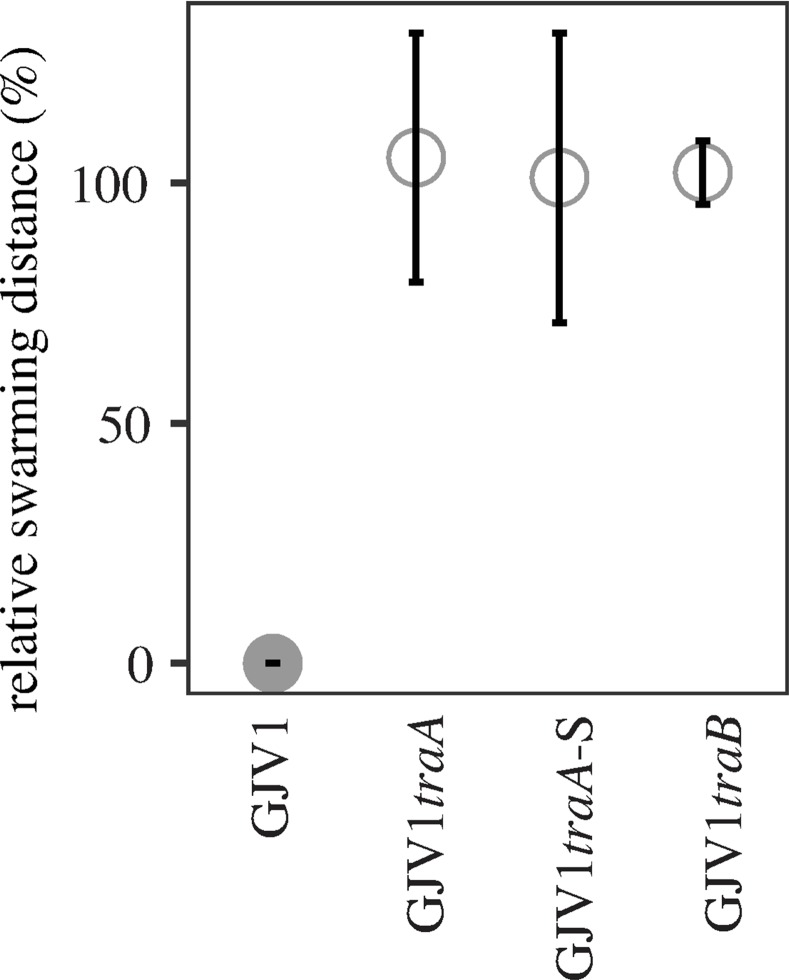
Disruption of *traA* and *traB* alleviates inhibition of GJV1 swarming by DK101. When mixed at a 1:1 ratio, DK101 inhibits swarming by GJV1 but not GJV1*traA*, GJV1*traA*-S or GJV1*traB*. *y-*axis values indicate the percentage-distance swarmed by each focal strain indicated on the *x* axis in mixture with DK101 relative to its swarming distance in pure culture (see Methods for details). Error bars are 95% confidence intervals, *n* = 3 replicates.

GJV1 swarms much faster than DK101 on agar surfaces due to the absence of S-motility in DK101 (S1 Fig). Thus, if there were no interaction between these strains in mixed cultures spotted onto agar, the GJV1 subpopulation would swarm outward at a rate similar that of a GJV1 monoculture. However, in such DK101:GJV1 mixes, GJV1 fails to swarm outward (Fig 1). Because i) strain DK1622 is killed by DK101 due to OME-mediated toxin delivery [46], ii) the genome sequence of GJV1 (our lab version of DK1622) is identical to the published DK1622 sequence excepting five mutations [51], and iii) disruption of both *traA* and *traB* alleviate DK101 inhibition of GJV1 swarming (Fig 1), we infer that DK101 inhibits GJV1 swarming by the same OME-delivered toxin that inhibited swarming by the version of DK1622 used by Dey *et al*. (2016) [46].

In contrast, mutants of GJV1 in which OME has been debilitated by disruption of *traA* or *traB* survive contact with DK101 and swarm outward at the same rate as pure colonies of the respective mutants (Fig 1). Two plasmids containing 788-bp and 543-bp fragments of *traA* (pCR_GJV1*traA* and pCR_GJV1*traA*-S, respectively, Table 2) were utilized to disrupt *traA*. The shorter fragment was used in a previous study [34] and we thus use it as a control to demonstrate elimination of OME by disruption with the longer fragment. (Allele-specific versions of the same long fragment were used to disrupt *traA* in natural isolates for the experiments reported below (Table 2).) Mutants with *traA* disrupted by both the short and long fragments were equally able to escape swarm inhibition by DK101 (Fig 1), implying that both plasmids debilitate OME.

### A CMI between DK101 and GJV1 does not require functional TraA

**Fig 2:**
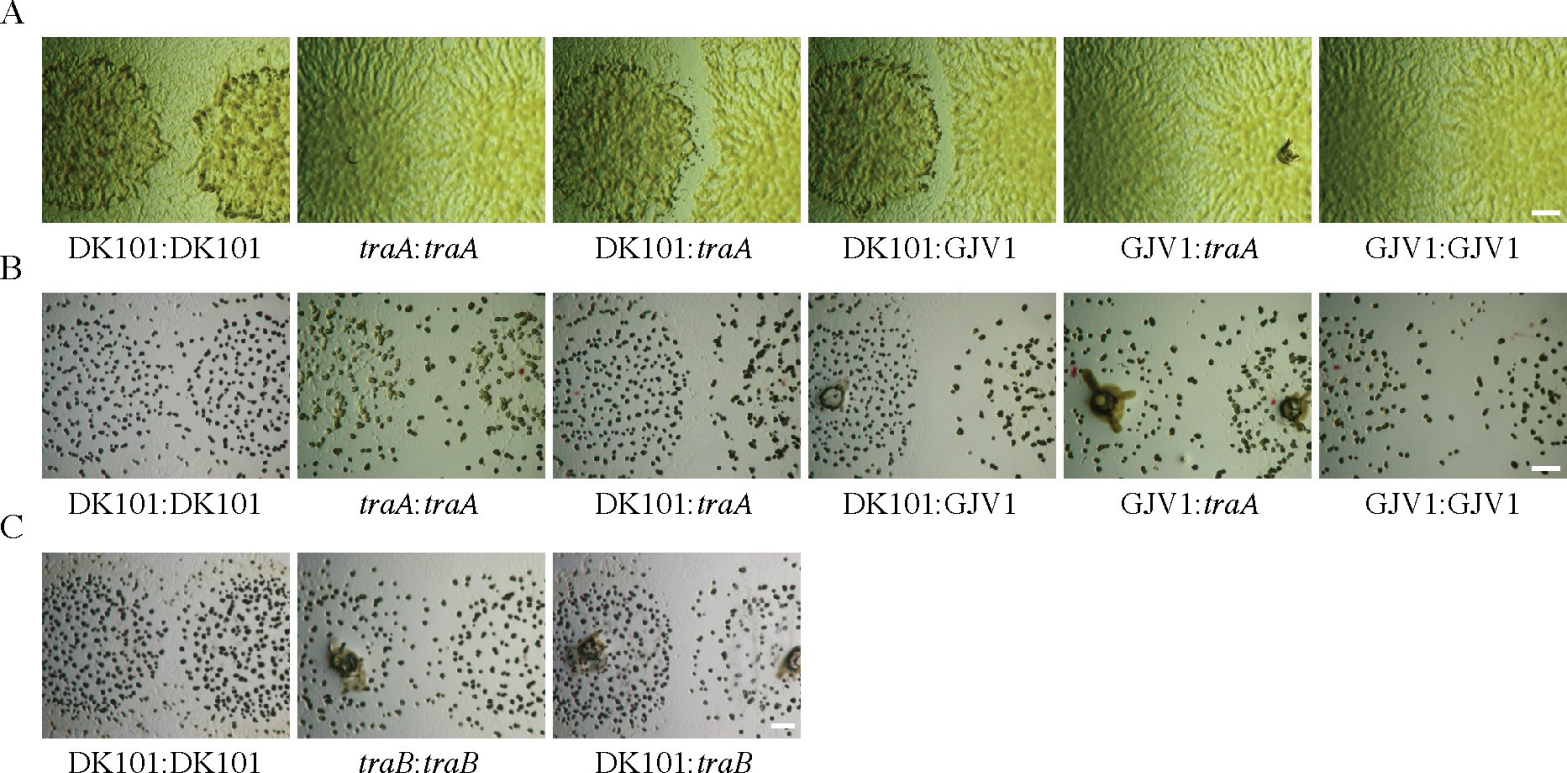
Disruption of *traA* or *traB* does not eliminate a CMI between GJV1 and DK101. Colony-encounter phenotypes in pairings of GJV1, GJV1*traA* (abbreviated to ‘*traA’* below images), and DK101 on 0.3% (**A**) and 0.1% (**B**) casitone media. For both **A** and **B,** colonies merge freely in self-self encounters of GJV1, GJV1*traA* and DK101 and in the non-self encounter of GJV1 and GJV1*traA*. In the left-most image of row **A**, the two DK101 colonies have darker center regions surrounded by distal regions that are less opaque but are nonetheless covered by cells. In this image, DK101 cells are continuous across the entire colony-interface region and do not exhibit a CMI phenotype. In contrast, strong CMI demarcations are formed in non-self encounters between DK101 and both GJV1 and GJV1*traA*. **C**. Interfaces between GJV1*traB* (abbreviated to ‘*traB’* below images) and DK101 colonies growing on 0.1% casitone also exhibit a clear CMI whereas self-self interfaces do not. Scale bars: 1 mm.

Colonies of DK101 and GJV1 on the same agar plate form a clear CMI boundary upon encounter on both nutrient-rich and nutrient-poor agar (Fig 2A and 2B, respectively). This CMI might have been caused by the same mechanism of OME-mediated killing of GJV1 by DK101 observed when these strains are homogeneously mixed. Under this hypothesis, disruption of *traA* in either strain should eliminate (or greatly reduce) boundary formation, just as disruption of *traA* alleviates killing of GJV1*traA*. However, we observed that disruption of *traA* in GJV1 did not visibly reduce the CMI between GJV1*traA* and DK101 relative to GJV1 (Fig 2A and 2B). Thus, the CMI between these two closely related laboratory strains does not appear to involve OME-mediated toxin delivery. Disruption of *traB* also failed to eliminate the CMI between DK101 and GJV1 (Fig 2C).

### CMIs between TraA-homotypic natural isolates also do not require functional TraA

Eleven of the 78 Tübingen cm-scale natural isolates sampled by Vos & Velicer [54] were selected for *traA* disruption (Table 1) to test whether TraA-mediated toxin transfer is commonly responsible for kin discrimination between TraA-homotypic strains in a local natural population. These isolates tend toward antagonistic interactions in pairwise mixes [19] and represent multiple TraA “recognition groups” [3]–sets of highly similar TraA sequences that allow OME to occur when paired across adjacent cells. In a previous study, these recognition groups were not found to predict CMI kin-discrimination types [3]. Plasmids carrying the same 788-bp *traA* fragment corresponding to that in pCR_GJV1*traA* were integrated into eleven natural isolates representing five *traA* alleles, which in turn are predicted to represent five TraA PA14 OME recognition groups (Table 2 in [3] and Figs 2 and 4 in [3]). Colony-encounter phenotypes were then compared for each parental natural isolate and its corresponding *traA* disruption mutant. This was done for pairings of each parent and its *traA* mutant with all other isolates examined here sharing the same *traA* allele.

**Fig 3:**
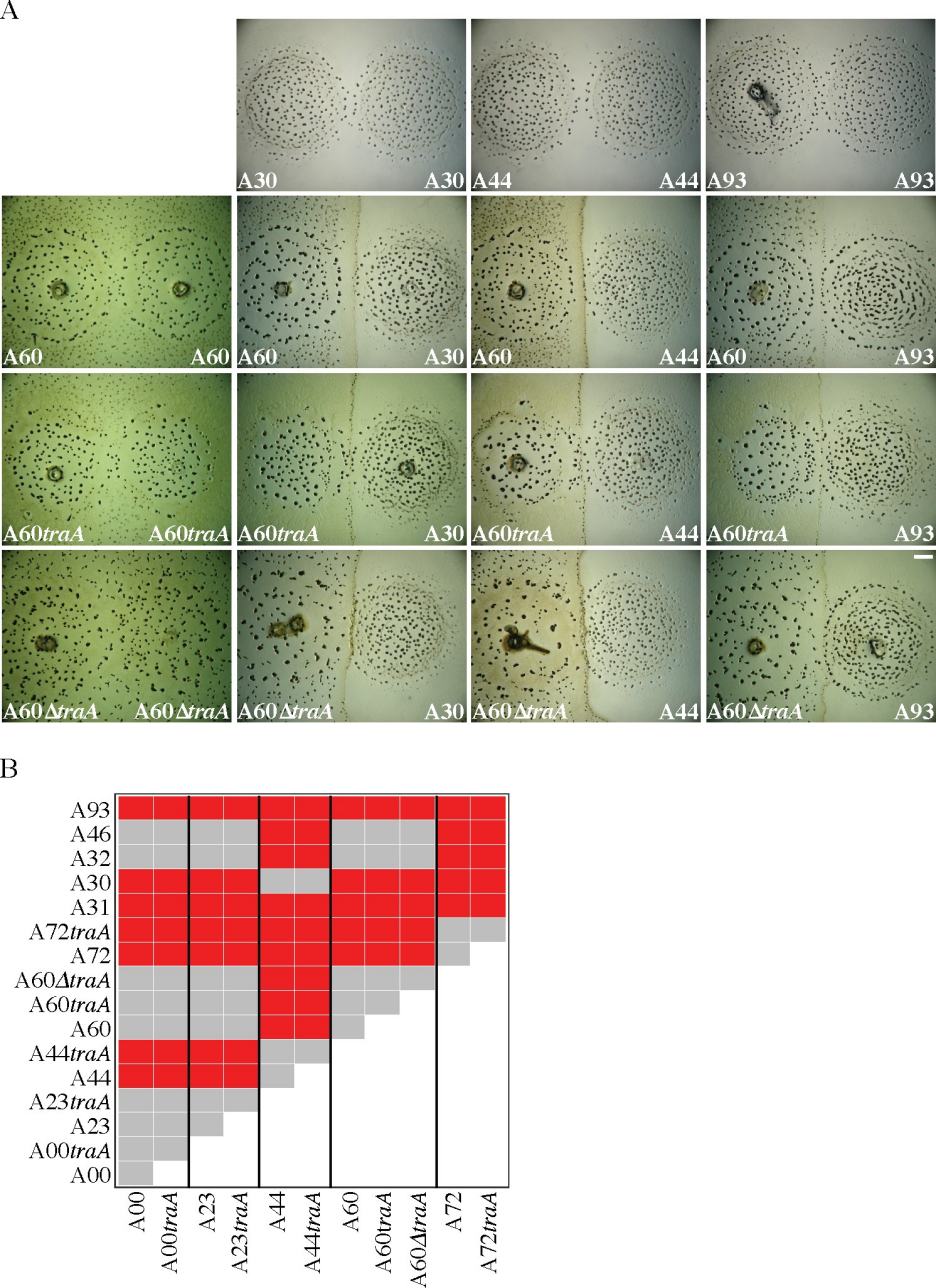
Disruption of *traA* does not alter CMI-occurrence patterns across pairings of *traA-*identical natural isolates. **A.** Examples of self-self colony-encounter phenotypes (left column and top row) and CMI phenotypes between distinct natural isolates and respective *traA* mutants (other images). Scale bar: 1 mm. **B.** All possible pairwise encounters between strains sharing the same *traA* allele for one of several alleles. (See also S2 Fig for results from other allele categories.) Red represents formation of visible CMI demarcation boundaries while grey represents the absence of such boundaries. In no case did disruption of *traA* (or deletion in the case of A60) eliminate a CMI boundary present between colonies of two natural isolates (or generate such a boundary not present between two isolates). The plasmid-integration and in-frame-deletion mutants of A60 exhibit CMIs with the same set of natural isolates as the parent strain.

None of the *traA* disruption mutants exhibited a different CMI-occurrence pattern in encounters with other natural isolates than their respective parental strains (Fig 3 and S2 Fig). This result indicates that TraA-mediated OME toxin transfer is not necessary for expression of CMI phenotypes in the examined set of strain pairs. Additionally, the CMI phenotypes involving the mutants were in no case visibly reduced relative to those of the respective parental strains (e.g. Fig 3A), suggesting that OME toxin transfer does not contribute significantly (or at all) to the observed CMIs.

The plasmid pCR_GJV1*traA* eliminates TraA function upon integration into GJV1 (Fig 1). It is expected that integration of similar plasmids that differ only in their *traA* allele debilitates *traA* function in all strains. Nonetheless, it is possible, if unlikely, that the non-disrupted 3’ portion of *traA* remaining after integration of pCR_*traA* (which includes the entire PA14-encoding region) might somehow retain function and allow OME to occur only in the mutants of natural isolates and not in GJV1*traA*. To address this possibility, we generated an in-frame deletion of a large segment of *traA* in a natural isolate (A60) corresponding to removal of almost the entire gene (amino-acid residues 13–695 from the 719 aa-long TraA sequence), including the entire PA14 region [34, 36]. We then compared CMI-boundary-occurrence patterns in pairings with other natural isolates for A60, A60*traA* and A60Δ*traA*. Like plasmid disruption of *traA* in A60*traA*, deletion of almost the entire *traA* sequence in A60Δ*traA* did not eliminate or visibly reduce the CMI phenotypes exhibited by strain pairs that include A60 (Fig 3).

### Inhibition of natural-isolate swarming by DK101 is *traA*-independent

**Fig 4:**
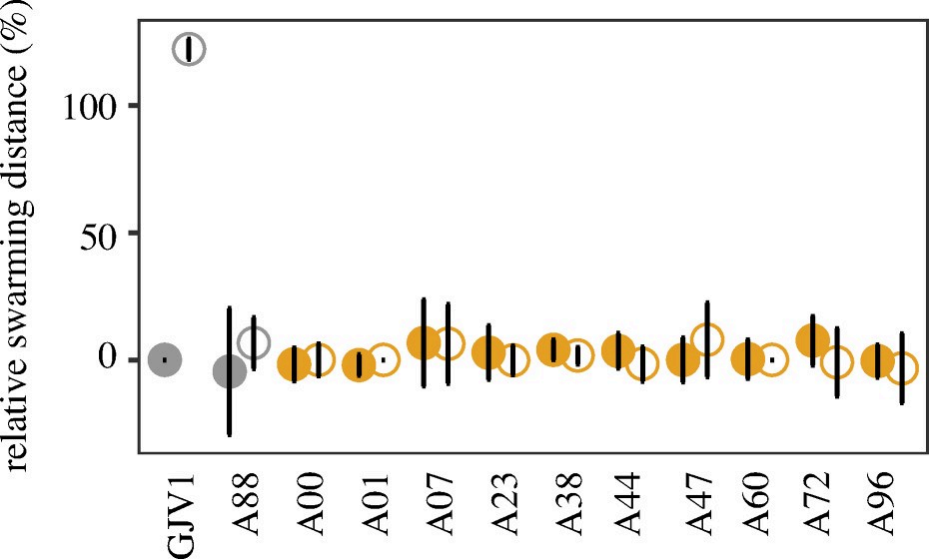
Disruption of *traA* does not alleviate DK101 inhibition of swarming in natural isolates. The *y* axis reflects the same relative swarming-distance parameter as in Fig 1. Two strains known (GJV1) or predicted (A88) to be compatible with DK101 for OME at *traA* are depicted in grey, whereas strains predicted to be incompatible with DK101 for OME are depicted in orange. Parental strains and their corresponding *traA* mutants are depicted by closed and open circles, respectively. All strains were mixed with DK101 at a 1:9 initial ratio. Error bars show 95% confidence intervals, *n* = 3 replicates.

As shown above, DK101 inhibits swarming by GJV1 in mixed cultures in an OME-dependent manner (Fig 1). Additionally, DK101 also causes OME-independent CMI phenotypes during colony encounters with GJV1*traA* (Fig 2), even though DK101 does not kill or reduce the swarming of that mutant in mixed cultures (Fig 1). To investigate other OME-independent social incompatibilities involving DK101, we tested whether DK101 exhibits *traA*-independent antagonisms toward several of the natural isolates examined here, all but one of which (A88) are predicted to be incompatible with DK101 for OME due to TraA dissimilarity [3, 36], as well as corresponding *traA* mutants. DK101 strongly inhibited swarm expansion by all of the natural isolates and corresponding *traA* mutants examined (Fig 4), demonstrating that DK101 exerts strong *traA*-independent toxicity toward all of these strains, including A88.

### DK101 and GJV1 kill a natural isolate without TraA-mediated delivery of Sit toxins

**Fig 5:**
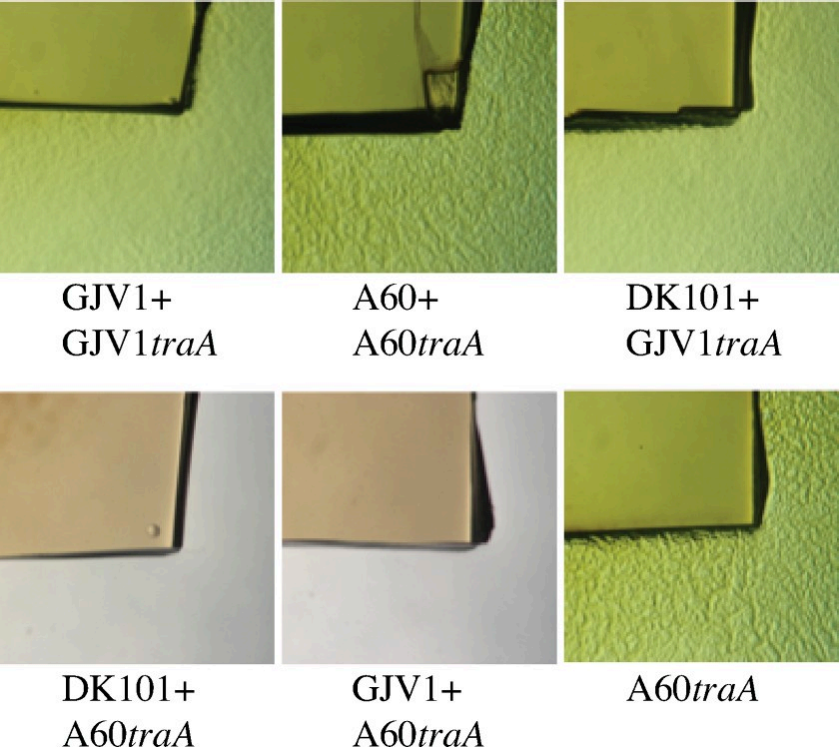
Inhibition of A60*traA* swarming by DK101 and GJV1 is caused by TraA-independent killing. Mixes of kanamycin-resistant *traA* mutants with kanamycin-sensitive non-mutants were incubated on CTT agar without kanamycin and subsequently transferred on onto CTT agar with kanamycin. Lack of swarming out-growth on the kanamycin plates indicates killing of the focal *traA* mutant by the focal non-mutant prior to exposure to kanamycin. None of the *traA* mutants are killed by its parental strain and GJV1*traA* is not killed by DK101 (top row). A60*traA* is killed by both DK101 and GJV1 (bottom row).

The severity of swarming inhibition exerted by DK101 on various natural isolates and their *traA* mutants suggested that the corresponding antagonisms mediated by DK101 may be lethal. To test this for one natural isolate, we spotted cultures of GJV1*traA* and A60*traA* each mixed with DK101 and its own parental strain on CTT hard agar. After four days of incubation, an agar section containing the entire colony was cut out and transferred upside-down onto CTT kanamycin-agar plates. Because the *traA* mutants are kanamycin-resistant (whereas DK101 is kanamycin-sensitive), they will grow and swarm outward after placement on kanamycin plates if DK101 does not kill them. GJV1*traA* survived interaction with DK101 and swarmed outward on kanamycin agar (Fig 5), whereas A60*traA* did not exhibit detectable growth or swarming. Thus, DK101 appears to secrete toxins lethal to A60*traA* that are not transferred by OME and which are responsible for the swarming-inhibition of A60 and A60*traA* (and likely other natural isolates as well) shown in Fig 4.

Additionally, because DK101 and GJV1 are expected to be identical across most of their genomes [46, 51, 58], the two strains are likely to make most or all of the same OME-independent toxins. Indeed, A60*traA* was killed by GJV1 as well as by DK101, but not by its parental strain A60 (Fig 5), implicating a killing mechanism that does not involve OME-mediated delivery of Sit toxins.

### DK101/GJV1 killing of A60*traA* requires a T6SS

Many antagonistic interactions between bacteria have been attributed to the T6SS [22, 24, 59, 60]. The T6SS functions in a cell-contact dependent manner in which toxic effector proteins are transferred from donor to recipient cells and can kill recipients that do not produce an appropriate antitoxin [60, 61]. *M*. *xanthus* encodes the major T6SS gene cluster composed of thirteen loci [62] that include a primary *vgrG* gene (*vgrG1*), but also carries an additional orphan *vgrG* paralog (*vgrG2*) located elsewhere in the genome [55]. VgrG proteins are typically structural components required for T6SS functionality and are located at the tip of the puncturing structure which is formed by Hcp hexamers [63, 64]. Previous work has shown that T6SS-dependent killing in *M*. *xanthus* is abolished if either the primary or orphan *vgrG* gene is disrupted [55]. Further, deletion of a putative effector gene (*tsxE*) downstream of *vgrG2* similarly abolished T6SS-mediated antagonisms. Complementation of *tsxE* restored the killing phenotype and deletion of the associated immunity gene *tsxI* rendered the mutant susceptible to killing by its parental strain.

**Fig 6:**
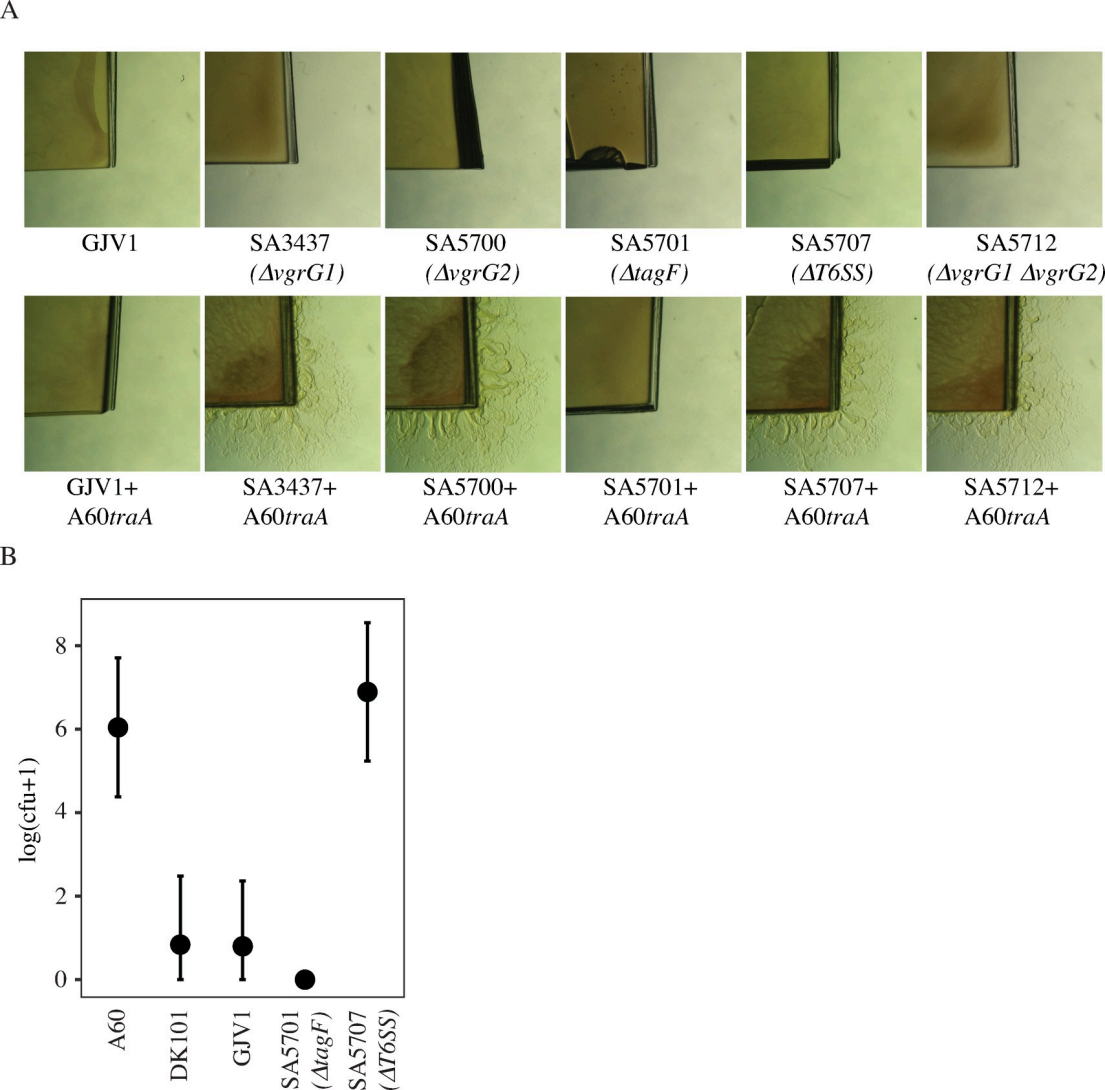
Deletion of T6SS genes alleviates TraA-independent killing of a natural isolate. **A.** Top row: Monocultures of kanamycin-sensitive GJV1 and T6SS mutants initially grown on CTT agar fail to grow when placed on CTT-kanamycin agar. Bottom row: Killing-assay phenotypes of cultures of A60*tra*A mixed with GJV1 and several T6SS mutants initially grown on CTT agar and transferred to CTT-kanamycin agar. GJV1 and a *ΔtagF* mutant (SA5701) both retain T6SS activity [55] and kill A60*traA*. Mutants known to have lost T6SS activity (SA3437, SA5700, SA5707, and SA5712 [55]) do not kill A60*traA*. **B.** CFU counts of A60*traA* after 24 hours of co-incubation with each strain indicated on the x axis. Error bars represent 95% confidence intervals, *n* = 3 replicates.

To test whether the observed TraA-independent killing of A60*traA* by GJV1 is mediated by its T6SS, we used five T6SS mutants created in the DK1622 background [55]. Four of these five mutants had their T6SS defect verified by Hcp secretion assays and complementation, whereas one (SA5701, *ΔtagF*) retained T6SS activity [55]. Killing was first assayed by determining the presence or absence of A60*traA* growth on kanamycin agar after co-culture with the T6SS mutants. Consistent with the results of Troselj *et al*. [55], we found that the *ΔtagF* mutant SA5701 retained the ability to kill *A60traA*, whereas the other four mutants, which each lacked one or both of the *vgrG* genes, were unable to kill *A60traA* (Fig 6A). These results are supported further by a quantitative assay of survival by *A60traA* after co-incubation with several strains, including its own parent A60. After 24 hrs of co-incubation at a 1:2 ratio, *A60traA* CFU counts were greatly reduced by DK101, GJV1 and the *ΔtagF* mutant SA5701, but not by the T6SS-deletion mutant SA5707, relative to the control mix of *A60traA* with A60 (Fig 6B).

**Fig 7:**
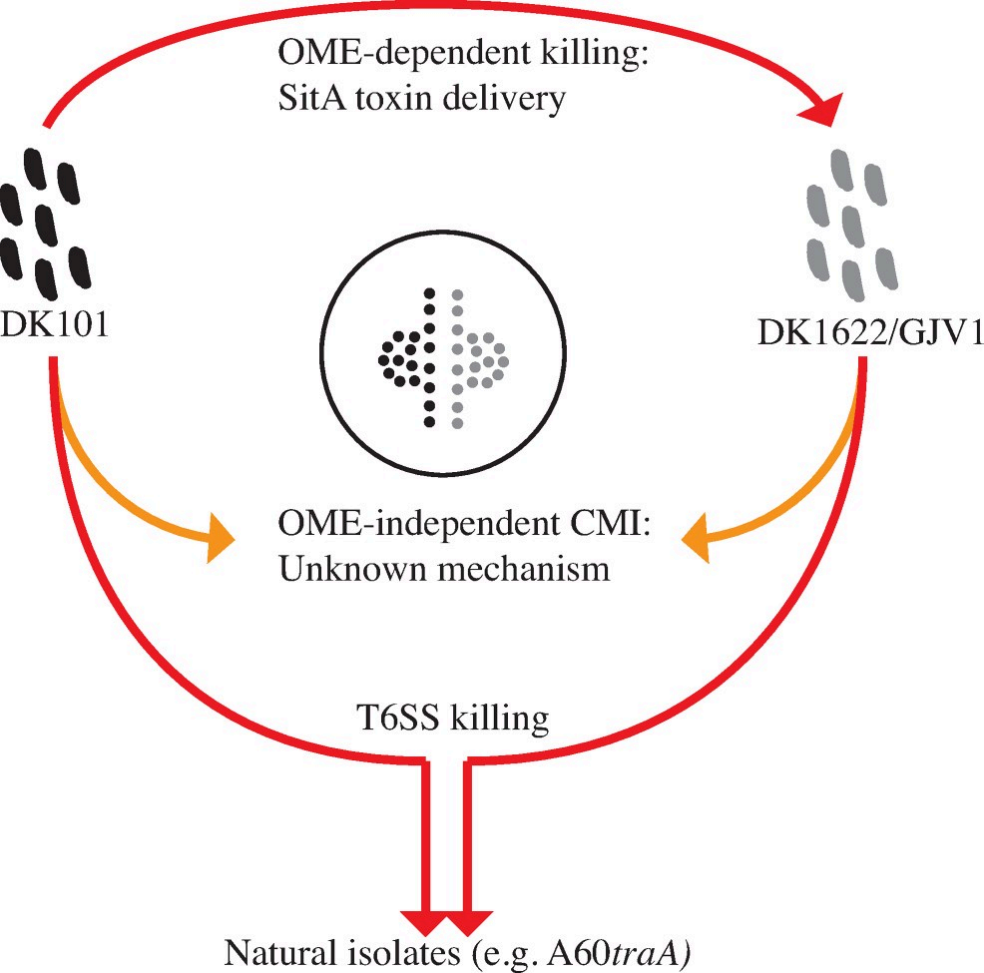
Model of known and proposed interactions and relationships among DK101, DK1622 and GJV1. DK101 kills DK1622 and GJV1 due to OME delivery of SitA toxins ([26, 46] and Fig 1). A CMI boundary forms between colonies of DK101 and GJV1 (and by inference DK1622) that is not caused by OME-delivered Sit toxins (Fig 2). DK101, DK1622 and GJV1 are proposed to kill (and thus inhibit swarming by) natural isolates (and *traA* mutants of such isolates) due to a shared T6SS (Fig 6).

These results implicate the DK1622/GJV1 T6SS as the mechanism by which GJV1 kills both A60 and A60*traA*. Further, from the expected overall genomic similarity of GJV1 and DK101 [46, 51], we infer that T6SS-dependent killing is most likely responsible for the TraA-independent swarming inhibition of the natural isolates by DK101 as well. A model of known and proposed interactions and relationships among DK101, DK1622 and GJV1 collectively emerging from our results and those of previous studies is presented in Fig 7.

### Developmental antagonisms between TraA-identical natural isolates are also independent of OME

When *M*. *xanthus* natural isolates are mixed at unequal frequencies, the minority strain is often strongly antagonized by the majority type, suffering greatly reduced population size or outright extinction [48]. We tested for a possible role of OME-delivered toxins such as SitA in mediating antagonisms between TraA-compatible natural isolates during starvation-induced development. To do so, we screened for developmental antagonisms exerted against rifampicin-marked variants of strains A23, A47 and A96 when they were mixed as the minority type in 1:99 ratios with the other unmarked strains in this study sharing the same *traA* allele (A00, A23, A30, A32, A46, A60, A72 and A93 mixed with A23rif^*R*^; A07, A26, A47 and A96 mixed with A47rif^R^; and A07, A26, A47 and A96 mixed with A96rif^R^). Strain pairs were homogeneously mixed, starved on TPM agar and viable spore production after five days of starvation was quantified for the total population and both individual strains.

**Fig 8:**
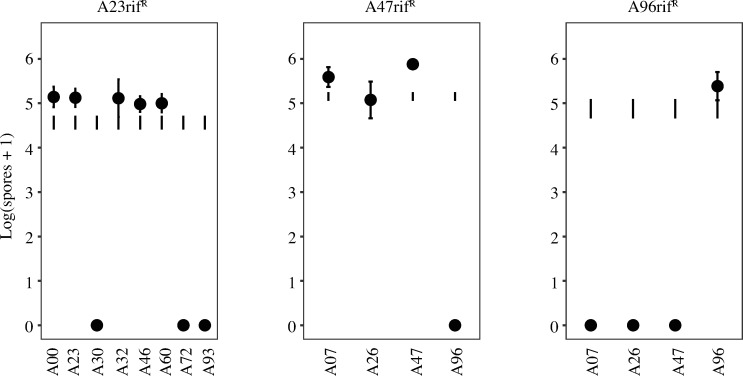
Antagonism patterns of focal minority strains in mixed groups during starvation-induced development. Actual spore production by marked natural isolates mixed in the minority at a 1:99 ratio with unmarked isolates (indicated on the *x* axis) prior to starvation-induced development is represented by closed circles. Expected spore production ranges of the minority strains based on pure-culture assays are depicted by error bars with no circles. Error bars represent 95% confidence intervals, *n* = 3 replicates.

In pure-culture controls, all strains examined produced high levels of viable spores (S3 Fig). In control pairings of the three rifampicin-resistant mutants of A23, A47 and A96 mixed as a minority with their unmarked parent, no antagonism was observed, as spore production by the minority marked type was not reduced by the parent relative to expectations from pure-culture assays (Fig 8). Among the 13 pairings of the rifampicin-resistant mutants with other (non-parental) strains, in seven cases the majority strain eliminated spore production by the minority strain (at our limit of detection) whereas in the other six pairings no antagonism was observed (Fig 8). Strains A30, A72 and A93 in the majority eliminated spore production by A23rif^R^, A96 similarly antagonized A47rif^R^ and A07, A26 and A47 antagonized A96rif^R^ (Fig 8).

**Fig 9:**
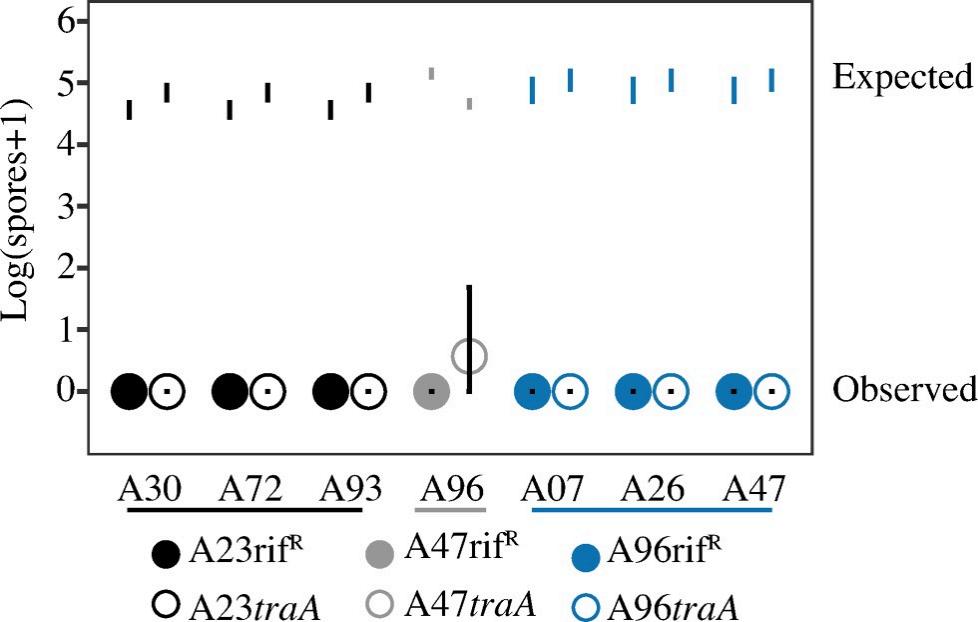
Severe antagonisms between TraA-identical natural isolates are independent of TraA. Antagonisms during development in which viable spore production by some natural isolates is eliminated (or nearly eliminated) in mixtures with other isolates are not alleviated by disruption of *traA*. Actual spore production by rifampicin-resistant variants of victim strains and respective kanamycin-resistant *traA* mutants in 1:99 initial mixtures with respective unmarked antagonizing isolates is represented by closed and open circles, respectively. Expected spore production of the victim strains from pure-culture assays is depicted by error bars with no circles. A23rif^R^ and A23*traA* (black circles) were each mixed with A30, A72 and A93, A47rif^R^ and A47*traA* (grey circles) were mixed with A96 and A96rif^R^ and A96*traA* (blue circles) were mixed with A07, A26 and A47. Error bars represent 95% confidence intervals, *n* = 3 replicates.

To examine whether OME-transferred toxins might fully or partially cause the observed developmental antagonisms, we tested whether the antagonisms are alleviated to any degree by disruption of *traA* in the parent of the respective minority victim strain. In all seven cases of severe antagonism, spore production by the corresponding *traA* mutant of the victim minority strain was also reduced to zero (or near zero) by the antagonistic majority strain, thus implicating OME-independent mechanisms (Fig 9).

## Discussion

The occurrence of five predicted TraA compatibility groups in a cm-scale *M*. *xanthus* population [3] (with multiple isolates representing each group) suggests that a large fraction of randomly selected isolate pairs will be incompatible for OME due to functional dissimilarity at the PA14 domain of TraA. For this reason alone, a large fraction of latent CMIs and antagonisms among all possible *M*. *xanthus* strain pairs (e.g. [19, 48, 65]) are expected to be independent of OME. Nonetheless, *M*. *xanthus* natural genetic diversity is highly structured spatially [47, 49], such that strains sampled at small spatial scales across which interactions between genotypes are most likely (e.g. μm–cm) may often be compatible for OME at TraA. It is thus relevant to test whether CMIs and antagonisms among TraA-compatible natural isolates of *M*. *xanthus* from the same local population generally involve OME-mediated toxin transfer, as this study has done.

The *traA* gene was disrupted in eleven *M*. *xanthus* natural isolates and potential alleviation of CMIs by *traA* disruption was examined for 33 isolate pairs. In no case were CMI phenotypes eliminated or even visually reduced by *traA* disruption (Fig 3 and S2 Fig). Moreover, seven severe antagonisms between distinct isolates identical at TraA were also not alleviated by *traA* disruption (Fig 9). Thus, our results do not support the hypothesis that OME-dependent toxin delivery is commonly responsible for inter-strain CMIs and antagonisms among natural isolates compatible for OME at TraA [26, 47]. Rather, these findings suggest that any CMIs and/or antagonisms among natural isolates caused primarily by OME-delivered toxins are rare relative to OME-independent mechanisms, even among strains compatible for OME at *traA*. In turn, these results do not strengthen the hypothesis that selection for resistance to newly evolved forms of OME toxin delivery is the primary evolutionary mechanism by which TraA has diversified into several types that are functionally incompatible for OME [3, 38].

Both the results of a previous study [3] and our findings here indicate that OME-independent CMIs can evolve rapidly. First, they are common among strains in the focal Tübingen cm-scale population [3] that are genomically similar yet TraA-incompatible. Second, we discovered that the very closely related lab strains GJV1 and DK101 form CMI boundaries between them, regardless of whether or not both strains have a functional copy of *traA* (Fig 2) and despite swarming inhibition of GJV1 by DK101 being alleviated by *traA* disruption (Fig 1). These strains differ primarily in the absence in GJV1 of a substantial portion of a highly variable 150 kb region (localized near position 2.2 Mbp in the genome of reference strain DK1622; [47]) in which phage genes are overrepresented and homologs of contact-dependent growth inhibition genes were found [47]. Partial deletion of this region in the lineage from DK101 to DK1622 (the immediate precursor to GJV1), including deletion of two of the three Mx-alpha prophage copies present in DK101 [46], may have generated not only the OME-dependent susceptibility of DK1622 to an Mx-alpha toxin produced by DK101, but also the OME-independent CMI that we observed between these strains (Fig 2). Finally, in a previous study, TraA-independent CMIs were found to evolve indirectly between a majority of pairs of lab-evolved *M*. *xanthus* populations that recently diverged from a common ancestor [1].

Future identification of genes responsible for CMIs and inter-strain antagonisms will be of interest for gaining a molecular-level understanding of intra-specific social divergence in natural populations of myxobacteria. Such antagonistic compounds might be secondary metabolites [66] or proteins, including bacteriolytic enzymes [67, 68] which have been suggested to aid in predation and act as anti-competitor agents [69–71] and T6SS effector toxins [28, 55]. Some anti-competitor compounds might be delivered by outer-membrane vesicles, which have been implicated in predation by *M*. *xanthus* [72]. The full range of OM-vesicle contents in this species is not known, but many hydrolases and proteases have been identified [73, 74]. While the binding of *M*. *xanthus* outer-membrane vesicles to other *M*. *xanthus* cells has not been demonstrated, it is thought that outer-membrane vesicles in general are able to fuse with other gram-negative bacteria [75, 76].

The T6SS is common among bacteria and is also found in multiple species of myxobacteria [55]. In this study, we found that T6SS-defective mutants of the lab reference strain DK1622 are unable to kill a natural isolate that is killed by both DK101 and GJV1, implicating their shared T6SS in these lethal interactions (Fig 6). Intraspecific variation in effector/immunity gene combinations can generate many mutually toxic T6SS types within the same bacterial species [77], a possibility that may explain some of the lethal antagonisms among *M*. *xanthus* natural isolates [48].

Wielgoss *et al*. 2016 [47] found a correlation between gene-content patterns in the 150 kb highly variable genomic region containing the prophage Mx alpha in DK101 and DK1622 (46) and previously categorized inter-colony compatibility types (or “allorecognition types”) [19]. This correlation suggested that variation in toxin-gene content in this region (e.g. encoding Sit, T6SS, and Rhs/CdiA toxins) may be responsible for at least some antagonisms among the Tübingen cm-scale strains, whether by TraA-dependent or TraA-independent mechanisms [47]. Such polymorphic toxin systems, which often include T6SS and *rhs/cdiA* loci, are common among both bacteria and archaea that do not carry TraA homologs, which appear to be specific to the myxobacteria [38, 78, 79].

We observed that the pattern of severe developmental antagonisms across strain pairings demonstrated in this study (Fig 8) corresponds exactly with the pattern of colony incompatibility documented by Vos & Velicer (2009) [19] among the respective (unmarked) strains (Table 3). The combination of this correspondence and the previously noted correlation of the colony-compatibility pattern in [19] with gene-content in the highly polymorphic genomic region [47] suggests that variation in toxin/immunity gene content in this region may be largely responsible for severe antagonisms among the Tübingen cm-scale isolates. This hypothesis requires further testing, but if it is the case, the results shown in Fig 9 suggest that the relevant toxins expressed from the 150 kb polymorphic region do not generally require TraA-dependent OME to reach and kill competing genotypes.

**Table 3:** Patterns of developmental antagonism and colony incompatibility.

Developmental competition strains		Antagonism of minority strain	Demarcation line between parental strains in [19]	CMI between parental strains in this study (Fig 3B and S2A Fig)
Minority	Majority			
A23rif^R^	A00	-	-	-
A23rif^R^	A30	+	+	+
A23rif^R^	A32	-	-	-
A23rif^R^	A46	-	-	-
A23rif^R^	A60	-	-	-
A23rif^R^	A72	+	+	+
A23rif^R^	A93	+	+	+
A47rif^R^	A07	-	-	-
A47rif^R^	A26	-	-	-
A47rif^R^	A96	+	+	+
A96rif^R^	A07	+	+	+
A96rif^R^	A26	+	+	+
A96rif^R^	A47	+	+	+

Our results do not exclude the possibility that OME-delivered Sit toxins contribute to some antagonisms and CMIs between TraA-compatible myxobacterial strains in nature. However, the finding that *traA* disruption does not alleviate any of the severe antagonisms examined this study suggests that the respective aggressor strains either do not produce toxins delivered by OME or, if they do, any effects of such TraA-dependent toxins are secondary to TraA-independent mechanisms. These results thus magnify the open question of how much fitness benefits from OME-mediated interference competition contribute to the evolutionary maintenance of the *traA/traB* operon in many species of myxobacteria [38] relative to other selective forces.

## Supporting information

S1 Fig GJV1 and DK101 differ in their monoculture swarming rates. In monoculture, GJV1 swarms faster than DK101 due to the motility defect of DK101. *y-*axis values indicate the swarming rate (mm/day) for each strain indicated on the *x* axis. Error bars are 95% confidence intervals, *n* = 3 temporally independent replicates.
(TIF)

S2 Fig Disruption of *traA* does not alter CMI-occurrence patterns across pairings of *traA*-identical natural isolates. All possible pairwise encounters between strains sharing the same *traA* allele for three different alleles representing three predicted TraA compatibility groups (**A, B,** and **C**, respectively**)** (3). Red represents formation of visible CMI demarcation boundaries, grey represents the absence of such boundaries, and black represents inconsistent results between replicates. In no case did disruption of *traA* eliminate a CMI boundary present between colonies of two natural isolates (or generate such a boundary not present between two isolates).
(TIF)

S3 Fig Monoculture spore production values of a subset of natural isolates. Viable spore production of A23, A47, A96, their respective rifampicin-resistant variants and kanamycin-resistant *traA* mutants, and all other isolates mixed with A23, A47 or A96 during co-development (Fig 8). *y-*axis values show the log-transformed spore production of each isolate indicated on the *x* axis. Bars are colored either orange or grey to indicated predicted TraA compatibility. Error bars are 95% confidence intervals, *n* = 3 replicates.
(TIF)
