# Correction: Kin discrimination and outer membrane exchange in *Myxococcus xanthus*: Experimental analysis of a natural population

**DOI:** 10.1371/journal.pone.0228697

**Published:** 2020-01-30

**Authors:** The PLOS ONE Staff

## Notice of republication

This article was republished on January 21, 2020, to correct errors in Table 3 that were introduced during the typesetting process. The publisher apologizes for this error. Please download this article again to view the correct version.

## References

[1] Cossey SM, Yu Y-TN, Cossu L, Velicer GJ (2019) Kin discrimination and outer membrane exchange in *Myxococcus xanthus*: Experimental analysis of a natural population. PLoS ONE 14(11): e0224817 10.1371/journal.pone.0224817 31774841 PMC6880969

