# Supplementary figures and images for: Kin discrimination and outer membrane exchange in Myxococcus xanthus: Experimental analysis of a natural population
Source: PLoS One. 2019 Nov 27;14(11):e0224817. doi: 10.1371/journal.pone.0224817 (PMC6880969; doi:10.1371/journal.pone.0224817)

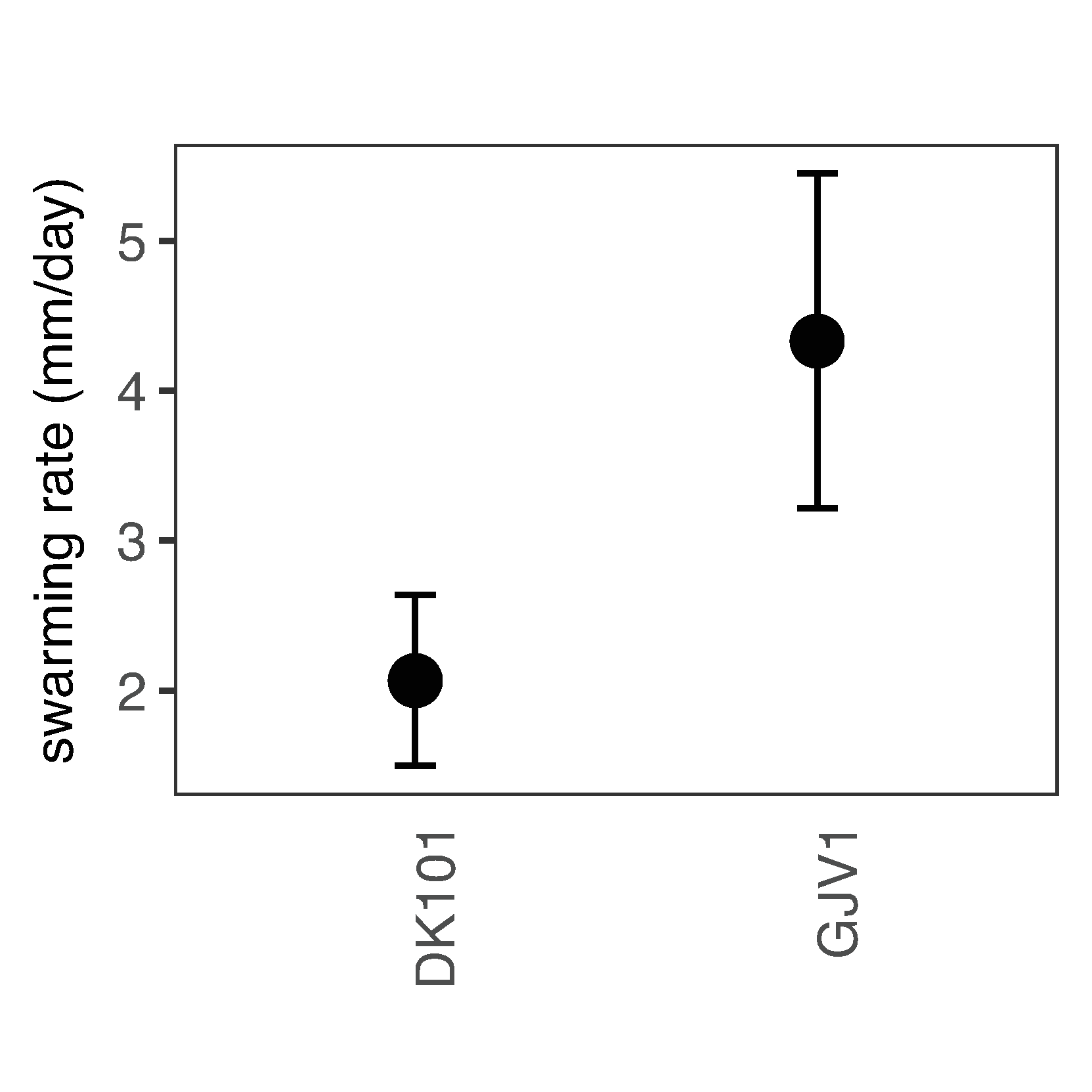

Supplement: S1 Fig — In monoculture, GJV1 swarms faster than DK101 due to the motility defect of DK101. y-axis values indicate the swarming rate (mm/day) for each strain indicated on the x axis. Error bars are 95% confidence intervals, n = 3 temporally independent replicates. (TIF) [file pone.0224817.s001.tif]

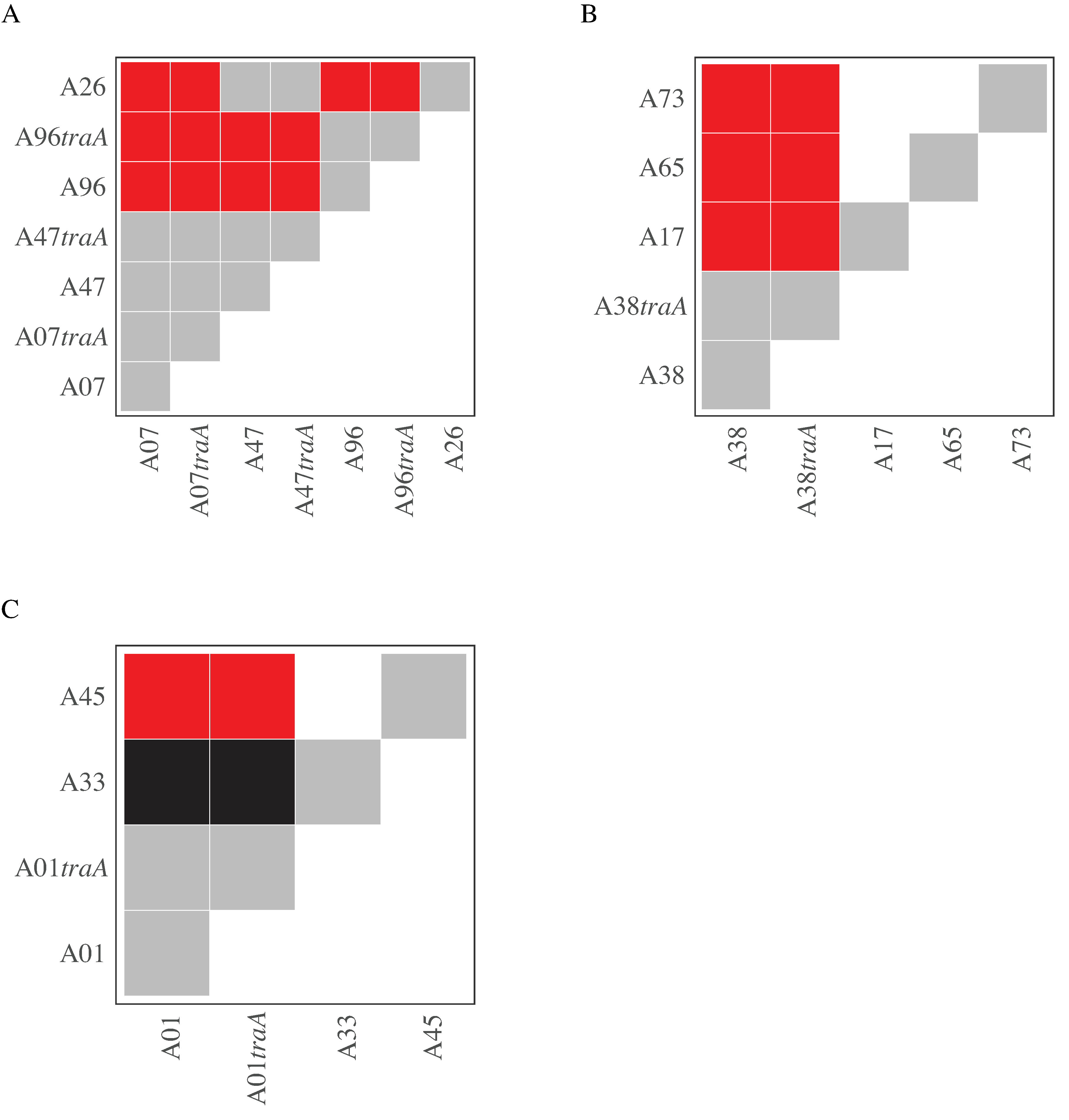

Supplement: S2 Fig — All possible pairwise encounters between strains sharing the same traA allele for three different alleles representing three predicted TraA compatibility groups (A, B, and C, respectively) (3). Red represents formation of visible CMI demarcation boundaries, grey represents the absence of such boundaries, and black represents inconsistent results between replicates. In no case did disruption of traA eliminate a CMI boundary present between colonies of two natural isolates (or generate such a boundary not present between two isolates). (TIF) [file pone.0224817.s002.tif]

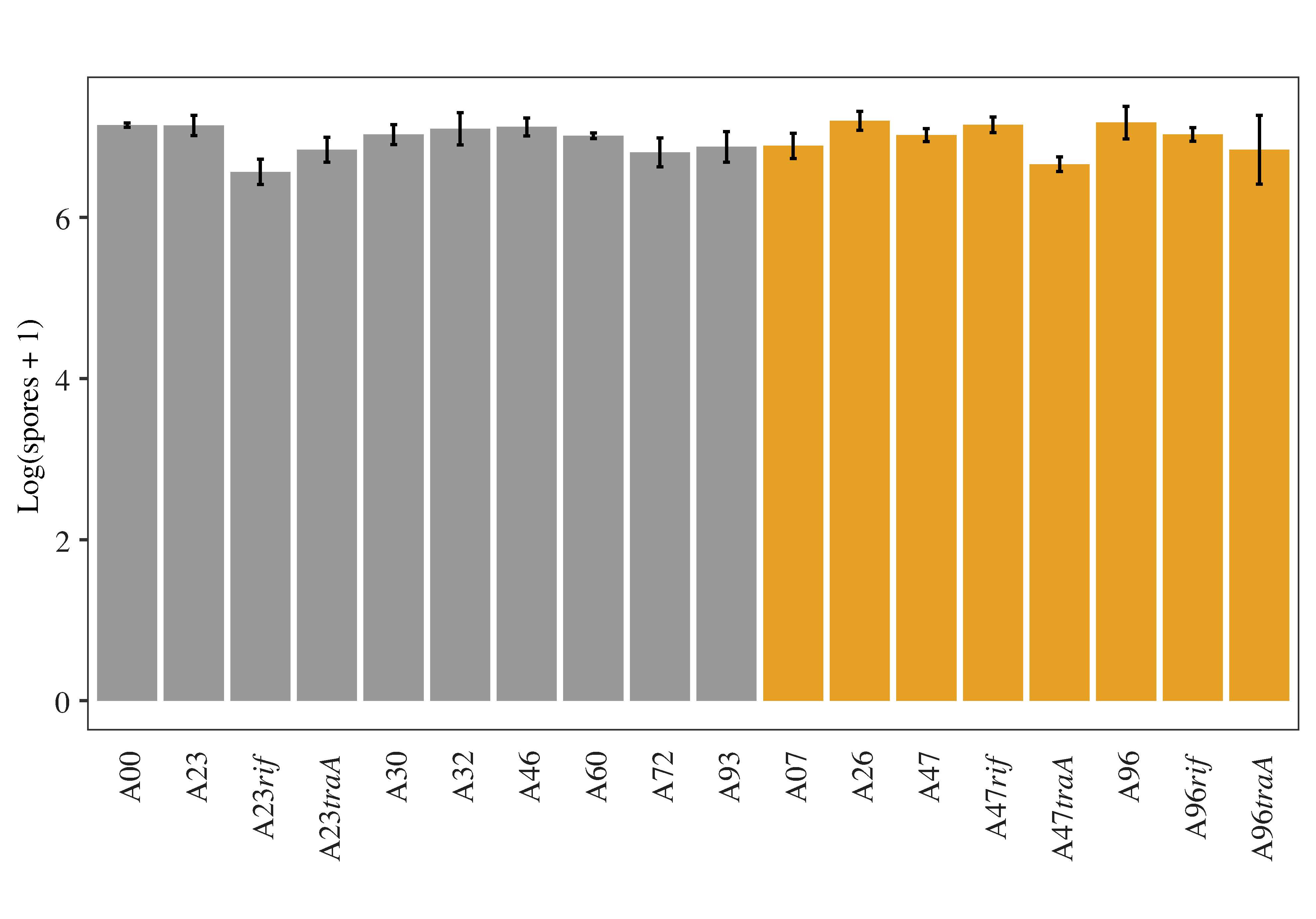

Supplement: S3 Fig — Viable spore production of A23, A47, A96, their respective rifampicin-resistant variants and kanamycin-resistant traA mutants, and all other isolates mixed with A23, A47 or A96 during co-development (Fig 8). y-axis values show the log-transformed spore production of each isolate indicated on the x axis. Bars are colored either orange or grey to indicated predicted TraA compatibility. Error bars are 95% confidence intervals, n = 3 replicates. (TIF) [file pone.0224817.s003.tif]
